# The Management of *Staphylococcus aureus* Bacteremia in the United Kingdom and Vietnam: A Multi-Centre Evaluation

**DOI:** 10.1371/journal.pone.0014170

**Published:** 2010-12-13

**Authors:** Guy E. Thwaites, United Kingdom Clinical Infection Research Group (UKCIRG)

**Affiliations:** Imperial College, London, United Kingdom; University of Cape Town, South Africa

## Abstract

**Background:**

*Staphylococcus aureus* bacteremia is a common and serious infection worldwide and although treatment guidelines exist, there is little consensus on optimal management. In this study we assessed the variation in management and adherence to treatment guidelines of *S. aureus* bacteremia.

**Methodology/Principal Findings:**

We prospectively recorded baseline clinical characteristics, management, and in-hospital outcome of all adults with *S. aureus* bacteremia treated consecutively over one year in eight centres in the United Kingdom, three in Vietnam and one in Nepal. 630 adults were treated for *S. aureus* bacteremia: 549 in the UK (21% methicillin-resistant), 80 in Vietnam (19% methicillin-resistant) and 1 in Nepal. In the UK, 41% had a removable infection focus (50% intravenous catheter-related), compared to 12% in Vietnam. Significantly (p<0.001) higher proportions of UK than Vietnamese patients had an echocardiogram (50% versus 28%), received more than 14 days antibiotic therapy (84% versus 44%), and received >50% of treatment with oral antibiotics alone (25% versus 4%). UK centres varied significantly (p<0.01) in the proportions given oral treatment alone for >50% of treatment (range 12–40%), in those treated for longer than 28 days (range 13–54%), and in those given combination therapy (range 14–94%). 24% died during admission: older age, time in hospital before bacteremia, and an unidentified infection focus were independent predictors of in-hospital death (p<0.001).

**Conclusions/Significance:**

The management of *S. aureus* bacteremia varies widely between the UK and Vietnam and between centres in the UK with little adherence to published guidelines. Controlled trials defining optimal therapy are urgently required.

## Introduction

*Staphylococcus aureus* bacteremia (SAB) is one of the most common serious bacterial infections worldwide. Its importance is well recognised in high income countries and over recent years much effort has been expended on disease surveillance and prevention, particularly for infections caused by methicillin-resistant *S. aureus* (MRSA). Despite this, *S. aureus* is the commonest cause of bloodstream infections in the US with the highest mortality[1]. In the UK there are more than 12,000 cases of SAB each year[2], of which around 30% die[3].

Recent studies from South East Asia have also documented *S. aureus* as a significant and under-recognised cause of community and hospital-acquired sepsis[4], [5], [6], [7]. These studies suggest similar clinical manifestations of SAB to high income countries, with MRSA causing a considerable burden of disease, but mortality may be higher[4]. There are, however, few data comparing treatment and outcomes following SAB in these different settings.

Current treatment guidelines suggest that SAB should be treated with prolonged intra-venous (IV) therapy: a minimum of 14 days for those with uncomplicated disease, and 4–6 weeks for those with a deep focus of infection[8], [9], [10], [11]. A penicillinase-stable beta-lactam antibiotic (e.g. flucloxacillin, oxacillin, nafcillin) is recommended to treat methicillin-susceptible SAB; and a glycopeptide (e.g. vancomycin or teicoplanin) is recommended for MRSA infections, or if the patient is intolerant of beta-lactams. Combination therapy (e.g. beta-lactam/glycopeptide *plus* gentamicin or rifampicin) is generally not recommended in these guidelines, except in severe MRSA infections (e.g. endocarditis, prosthetic joint infections). These recommendations, however, are based on data from fewer than 1500 patients randomised in 16 controlled trials. Much current practice is therefore based on clinical experience and observational studies. Recent surveys of European and US physicians have revealed diverse opinions concerning optimal SAB management[12], [13], [14] and there is some evidence of substantial practice variation within these regions[15], [16].

Our aim was to document the clinical characteristics, management, and in-hospital outcome of adults with SAB across 8 centres in the UK, 3 in Vietnam and 1 in Nepal to investigate the degree of practice variation across the centres, adherence to current treatment guidelines, and baseline factors predicting outcome.

## Results

Data were recorded on 657 consecutive adults diagnosed with *S. aureus* bacteremia. In 27 (4%), the isolate was considered a contaminant (1 from Vietnam and 26 from the UK). All subsequent analyses are based on the remaining 630 patients: 549 from the UK (1 centre had 3 patients, 7 had 49–150); 80 from Vietnam and 1 from Nepal.

Patients in Vietnam/Nepal were younger, more likely to be intra-venous drug users (IDU), had longer duration of symptoms, were less likely to have an IV catheter-related infection and more likely to have endocarditis than UK patients (**table 1**).

**Table 1:** Baseline characteristics of patients enrolled, by country (N = 630).

Factors^1^	United Kingdom N = 549	Vietnam/Nepal N = 81	p-value
MRSA bacteremia	116 (21%)	15 (19%)	0.62
Male gender	355 (65%)	58 (72%)	0.23
Age at positive culture (years) Median (IQR)	63.7 (46.9–77.3)	35.6 (27.0–54.1)	<0.001
<40 years	91 (17%)	48 (59%)	<0.001
40–59 years	146 (27%)	16 (20%)	
60–79 years	198 (37%)	15 (19%)	
≥80 years	104 (19%)	2 (2%)	
Intravenous drug use	54 (10%)	21 (26%)	<0.001
Diabetes mellitus	111 (21%)	11 (14%)	0.11
Immune suppression^2^	236 (47%)	30 (38%)	0.15
Days from admission to positive blood culture:			
Before/same day	269 (50%)^3^	29 (36%)	0.004
1–2 days after	92 (17%)	19 (23%)	
3–6 days after	48 (9%)	23 (28%)	
7–13 days after	55 (10%)	5 (5%)	
≥14 days after	78 (14%)	5 (6%)	
Duration of symptoms before positive blood culture:			
≤24 hours	209 (48%)	1 (1%)	<0.001
>24–72 hours	125 (29%)	12 (17%)	
>72 hours	101 (23%)	58 (82%)	
Focus of infection:^4^			
Intra-venous catheter alone	113 (21%)	3 (4%)	<0.001
Other source of infection, and removable^5^	110 (20%)	6 (8%)	
Not removable^6^	213 (40%)	38 (48%)	
Not established	101 (19%)	33 (41%)	
Whether site was infected:^7^			
Central venous catheter	116 (22%)	3 (4%)	<0.001
Peripheral line	37 (7%)	2 (2%)	0.12
Implanted vascular device	10 (2%)	0 (0%)	0.37
Native heart valve	18 (4%)	12 (15%)	<0.001
Prosthetic heart valve	3 (1%)	1 (1%)	0.43
Native joint	26 (5%)	4 (5%)	0.99
Prosthetic joint	10 (2%)	0 (0%)	0.37
Vertebral bone	15 (3%)	2 (3%)	0.99
Other bone	17 (3%)	1 (1%)	0.49
Soft tissue	175 (34%)	24 (30%)	0.47
Thrombophlebitis	29 (6%)	1 (1%)	0.16

1 Values in parentheses are percentages of total with information available, unless otherwise stated. Column percentages may not sum to 100% due to rounding. Number of patients with missing data: susceptibility to methicillin (10), gender (1), age at positive culture (10), IV drug use (24), diabetes (31), immune suppression (51), days from admission to positive blood culture (7), duration of symptoms before positive blood culture taken (124).

2 Defined as any intrinsic (e.g. malignancy, chronic liver or kidney failure) or extrinsic (e.g. drugs) factor which might attenuate immune response as judged by the treating physician.

3 15 patients had date of positive blood culture before date of admission.

4 Excludes 11 patients without any information available on foci of infection, and 2 patients with foci of infection given (prosthetic heart valve and prosthetic joint) but not known if considered removable.

5 Whether or not the focus was removable was judged on a case-by-case basis by the attending physician. There was no study-wide definition.

6 3 patients had intra-venous catheter related infection which, in the individual clinical circumstances, were not considered removable.

7 Information was sought on whether individual sites were infected. The proportion of patients with a given site infected was based on those with information available. Patients could have more than one site infected.

Twenty-one percent (95% confidence interval (CI) 18–25%) of isolates from the UK were methicillin-resistant compared with 19% (11–29%) from Vietnam/Nepal. The range among UK centres was 17% to 26% (p = 0.81). The extended antibiotic susceptibility profiles for all the isolates are given in **table 2**.

**Table 2:** Antibiotic susceptibility test results of *S. aureus* isolates.

Antibiotic susceptibility^1^	MRSA	MSSA
Penicillin susceptible	0/131 (0%)	83/483 (17%)
Erythromycin susceptible	39/120 (33%)	393/462 (85%)
Gentamicin susceptible	112/126 (89%)	468/480 (98%)
Fucidic acid susceptible	95/113 (84%)	370/407 (91%)
Rifampin susceptible	125/127 (98%)	434/436 (99%)
Tetracycline susceptible	110/117 (94%)	403/423 (95%)
Ciprofloxacin susceptible	20/122 (16%)	415/444 (93%)
Mupirocin susceptible	81/88 (92%)	262/269 (97%)
Vancomycin susceptible	122/122 (100%)	444/444 (100%)
Teicoplanin susceptible	61/61 (100%)	207/211 (98%)
Linezolid susceptible	89/89 (100%)	316/316 (100%)

1 Denominator variation is due to differences between centres in tests being performed routinely.

### Infection focus: identification and removal

Information on focus of infection was available for 617 (98%) patients (**table 1**). Removable foci were removed in 92% of cases, a median of 2 days (inter quartile range (IQR) 0–4 days) from the first positive blood culture. Focus removal occurred before or on the same day as the blood culture in 33%, after 1–2 days in 31%, 3–6 days in 18%, 7–13 days in 11%, and after 2 weeks in 7%. There was no significant variation between the UK centres in the timing of focus removal (**table 3**).

**Table 3:** Comparison of factors relating to management of SAB between UK and Vietnam/Nepal, and between UK centres.

	Number of patients enrolled (N = 630)	Percentage (95% confidence interval) of patients						
		Underwent echocardiogram^1^	Treated before or within 1 day of positive culture^2^	Received oral antibiotics exclusively for >50% of time on treatment^3^	Treated for <14 days with oral or IV therapy^3^	Treated for ≥28 days with oral or IV therapy^3^	Received combination therapy if treated^4^	Removable focus removed before or within 2 days of positive culture
**Region**								
UK	549	50% (46–54%)	81% (77–84%)	25% (21–30%)	16% (13–20%)	32% (27–37%)	48% (43–52%)	59% (52–66%)^5^
Vietnam/Nepal	81	28% (19–40%)	86% (76–93%)	4% (0–13%)	56% (41–70%)	8% (2–19%)	47% (35–59%)	- 5
P-value comparing UK vs Vietnam/Nepal		<0.001	0.33	<0.001	<0.001	<0.001	0.86	
**UK centres** 6								
1	89	47% (36–58%)	79% (69–87%)	28% (17–40%)	26% (16–39%)	26% (16–39%)	51% (40–62%)	58% (41–74%)
2	49	40% (26–55%)	92% (80–98%)	27% (14–43%)	12% (4–26%)	34% (20–51%)	94% (83–99%)	80% (52–96%)
3	150	53% (45–62%)	88% (82–93%)	16% (9–24%)	17% (10–25%)	38% (29–48%)	40% (32–48%)	66% (51–79%)
4	77	63% (51–75%)	82% (71–90%)	19% (9–34%)	17% (7–31%)	31% (18–47%)	45% (33–57%)	48% (31–66%)
5	57	37% (24–51%)	58% (44–72%)	12% (2–30%)	8% (1–25%)	54% (33–73%)	51% (37–65%)	41% (22–61%)
6	71	49% (36–61%)	76% (64–85%)	40% (28–53%)	13% (6–23%)	13% (6–23%)	14% (7–25%)	62% (47–76%)
7	53	55% (40–68%)	82% (69–92%)	39% (24–57%)	13% (4–28%)	42% (26–59%)	67% (52–80%)	67% (35–90%)
P-value comparing UK centres		0.06	<0.001	0.003	0.28	0.002	<0.001	0.15

1 Excludes 22 patients missing information on whether had an echocardiogram.

2 Excludes 29 patients missing information on active anti-staphylococcal antibiotic treatment, and 2 patients missing date started treatment.

3 Based on 429 patients with duration of treatment recorded, excluding those who died on treatment or within 2 days of stopping therapy.

4 Excludes 6 patients known to have been treated but were missing information on whether received combination therapy.

5 217/223 patients in the UK with removable focus had information on whether focus was removed, and if removed, date of removal recorded. Information on whether focus was removed was available for 7 of the 9 patients in Vietnam/Nepal with removable focus, with focus removed within 2 days of positive culture in 4 patients.

6 Excludes 1 UK centre with 3 patients enrolled.

### Echocardiogram

Echocardiography was performed in 50% (264/527) of patients in the UK compared to 28% (23/81) in Vietnam/Nepal (p<0.001) (**table 3**). Data on the use of trans-oesophageal echocardiography, or the influence echocardiography on treatment decisions, were not recorded. 59% of UK patients with a focus which was not IV catheter-related but considered removable, had an echocardiogram; compared to 50% with a non-removable focus, 45% with an IV catheter-related infection, and 46% with no focus established (p = 0.13).

### Start and choice of antibiotic treatment

Active anti-staphylococcal antibiotic treatment was given to 99% (593/601) of patients. Eight were not treated, either because they had died (n = 4) or were discharged (n = 4) before treatment was started. 81% of patients were treated before or within 1 day of when the positive blood culture was taken; this proportion was similar in the UK and Vietnam/Nepal, but differed between UK centres (**table 3**).

Flucloxacillin was used to treat MSSA bacteremia in a similar proportion in the UK (85%) and Vietnam/Nepal (81%), but varied from 68% to 98% among UK centres (p = 0.003) (**table 4**). A glycopeptide was given in 89% (62/70) of cases of MSSA bacteremia who did not receive flucloxacillin. The reasons for not using flucoloxacillin/other beta-lactams in these patients were not recorded. Flucloxacillin was used empirically, before susceptibility test results, in 18% of MRSA bacteremia in the UK and 86% (12/14) in Vietnam/Nepal.

**Table 4:** Antibiotics given for the treatment of SAB at any time during treatment, including early empirical treatment (N = 578^1^).

Antibiotics^2^	MRSA		MSSA	
	UK (N = 105)	Vietnam/Nepal (N = 14)	UK (N = 402)	Vietnam/Nepal (N = 57)
Flucloxacillin	19 (18%)	12 (86%)	340 (85%)	46 (81%)
Vancomycin	71 (68%)	10 (71%)^3^	88 (22%)	19 (33%)
Teicoplanin	41 (39%)	0 (0%)	61 (15%)	0 (0%)
Linezolid	15 (14%)	0 (0%)	12 (3%)	0 (0%)
Rifampin	22 (21%)	1 (7%)	62 (16%)	3 (5%)
Aminoglycoside	22 (21%)	2 (14%)	71 (18%)	11 (19%)
Fucidic acid	6 (6%)	0 (0%)	40 (10%)	0 (0%)
Clindamycin	3 (3%)	0 (0%)	43 (11%)	0 (0%)

1 Excludes 15/593 patients known to have been treated but information missing on antibiotics received (n = 10) or susceptibility to methicillin (n = 5, all received flucloxacillin).

2 Of 578 with information on antibiotics received, between 1–4 had missing information on whether they received an antibiotic. Antibiotics other than those listed were given to 81/578 (15%) patients. The most commonly used were a fluoroquinolone (n = 28, 5%) and doxycycline (n = 12, 2%). Daptomycin and tigecycline were used once.

3 The 4 patients not treated with vancomycin either died (n = 1) or were discharged (n = 3) within 24 hours of the positive blood culture.

The antibiotic treatment of MRSA bacteremia varied substantially in the UK. Teicoplanin was never used by two centres, but was prescribed to most patients with MRSA bacteremia in two others (7/10 and 18/20 cases, respectively). Linezolid was used to treat 6/12 MRSA bacteremias in one centre, but never used (0/30) in another.

### Route of administration

Nearly all patients (98%) treated were given IV antibiotics for some or all of their treatment. Fourteen patients (13 from UK) were given oral antibiotics alone; all had MSSA bacteremia, 6 had uncomplicated soft tissue infections, 5 had IV catheter-related infections, 1 had bone infection, and two had no established focus of infection. 11/14 received oral flucloxacillin alone, 5 for less than 14 days, and all survived to discharge.

**Figure 1:**
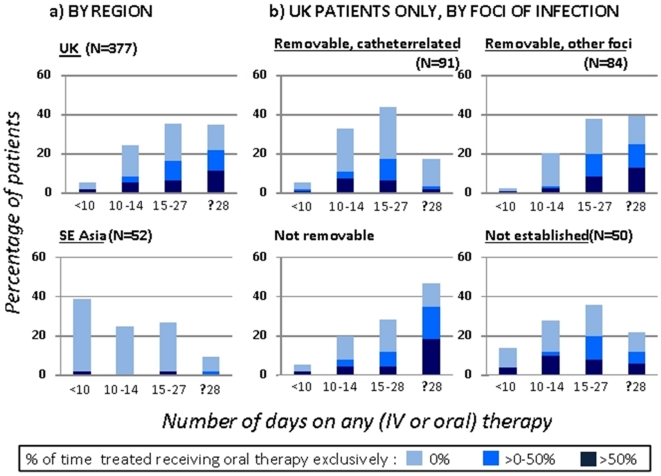
Duration on any therapy and proportion of time treated spent on oral therapy exclusively (N = 429^1^). ^1^ Excludes patients who died on therapy or within 2 days of stopping.

Patients were excluded from the duration of treatment analysis if they died during antibiotic therapy or within 2 days of stopping therapy (n = 108, 82% treated for <14 days), or if antibiotic start/stop dates were missing (n = 56). Among the remaining 429 patients, 49% (183/377) of patients in the UK received oral antibiotics exclusively for some part of their treatment, and 25% for more than half of their total treatment time (**figure 1** **;** **table 3**). In comparison, 6% (3/52) of cases in Vietnam/Nepal were treated with exclusively oral treatment at some point during therapy.

### Duration of antibiotic therapy

Of the 429 patients who started antibiotic treatment and did not die on or within 2 days of stopping therapy, 16% in the UK received <14 days of therapy (oral or IV) compared to 56% in Vietnam/Nepal (**table 3**). In the UK, 34% received <14 days of IV therapy, with significant variation between centres (range 10–56%, p<0.001).

In the UK, duration of therapy varied by focus of infection (p<0.001). The median days on therapy was 16 (IQR 14–19) for IV catheter-related infections; 22 (IQR 15–40) for infections caused by other removable foci; 27 (IQR 15–46) for non-removable infections; and 16 (IQR 13–26) if the focus was not established. The proportions receiving ≥28 days of therapy within each of these respective groups was 13%, 38%, 45% and 18% (**figure 1**). There was substantial variation between UK centres in the proportion on ≥28 days therapy (**table 3**), with differences remaining when restricted to patients with a focus which was removable but not catheter-related, or not removable (range 21% (8/39) to 60% (9/15); p = 0.03). Patients with no established focus were treated longer if they had MRSA bacteremia (median 24 days (IQR 14–36), n = 11) than MSSA bacteremia (15 days (13–22), n = 39; p = 0.03). In Vietnam/Nepal, the median duration of therapy was 13 days (IQR 6 to 22), with only 8% (4/52) treated for ≥28 days.

### Combination antibiotic therapy

Forty-eight percent of patients were treated at some point with combined use of two or more antibiotics. The proportion receiving combination therapy was similar for the UK (48%) and Vietnam/Nepal (47%), but varied substantially from 14% to 94% among UK centres (**table 3**). The proportion on combination therapy was similar for MRSA and MSSA bacteremia (43% and 48%, respectively; p = 0.37), but varied according to the focus. Combination therapy was used more if the focus was not removable (132/242, 55%), compared to either IV catheter-related infections (45/111, 41%), removable focus (not catheter-related) infections (50/112, 45%), or if a focus was not established (51/121, 42%) (p = 0.03). The choice of combination therapies used is summarised in **table 5**.

**Table 5:** Combinations of antibiotics received by patients with MRSA and MSSA bacteremia at any time during treatment (N = 274^1^).

Combination^2^	MRSA (N = 53) UK (N = 49) & Vietnam/Nepal (N = 4)	MSSA (N = 221)		
		Overall	UK(N = 193)	Vietnam/Nepal (N = 28)
Beta-lactam + aminoglycoside^3^	4 (8%)	60 (27%)	51 (27%)	9 (32%)
Glycopeptide + aminoglycoside^3^	15 (29%)	32 (15%)	30 (16%)	2 (7%)
Beta-lactam + rifampin^3^	0 (0%)	34 (16%)	33 (17%)	1 (4%)
Glycopeptide + rifampin^3^	20 (38%)	32 (15%)	32 (17%)	0 (0%)
Beta-lactam + fucidic acid^3^	1 (2%)	33 (15%)	33 (17%)	0 (0%)
Glycopeptide + fucidic acid^3^	3 (6%)	6 (3%)	6 (3%)	0 (0%)
Other combinations 4	26 (50%)	99 (45%)	78 (41%)	21 (75%)

1 Information on whether combination therapy was used was available for 587 of the 593 patients who were treated, of whom 279 (48%) received combination therapy. Of these, 274 had information on susceptibility to methicillin.

2 72/274 (26%) of patients received more than one type of combination during the same episode.

3 Information on whether a given combination was used was missing for 2–3 patients for each combination listed.

4 112 received another 2-drug combination; 13 received 3 or more drugs in combination. Of the 112 who received a 2-drug combination 34 received a glycopeptide + beta-lactam, 12 received a beta-lactam + fluoroquinolone, 12 received a beta-lactam + clindamycin, and 7 received a beta-lactam + macrolide. The remaining 47 received one of 22 different combinations.

### Adherence to current treatment guidelines

Treatment guidelines suggest that uncomplicated SAB should be treated with a minimum of 14 days antibiotics; those with a deep focus of infection (complicated infection) should receive 4–6 weeks of antibiotic therapy[8], [9], [10], [11]. In the UK, 74/94 (78.7%) of patients with uncomplicated SAB (defined as IV catheter-related, with prompt removal of the catheter and no other infection focus identified) received treatment which adhered to these guidelines (i.e. ≥14 days antibiotic therapy), although only 38.6% (72/186) with a deep, irremovable focus of infection received ≥28 days of antibiotic treatment. In Vietnam/Nepal 3 patients had uncomplicated SAB and one received ≥14 days antibiotic therapy; only 8.7% (4/46) of patients with complicated infection received ≥28 days of treatment.

Combination therapy is only recommended for severe MRSA infections, but was given to 47.8% (32/67) of patients with a deep irremovable MRSA infection, 52.6% (132/251) with a deep irremovable MSSA infection, and 41% (45/111) of all uncomplicated SAB secondary to a removed IV catheter.

### Outcome: metastatic complications and hospital discharge

Sixty seven (11%) patients (of 612) developed an additional site of infection, secondary to the primary focus and after the start of treatment; 18/129 (14%) with MRSA and 48/477 (10%) with MSSA bacteremia (susceptibility to methicillin was unknown in one patient). The commonest were lungs (n = 14, 21%) and soft tissues (n = 14, 21%), followed by native heart valves (n = 11, 16%) and bones/joints (n = 11, 16%). Metastatic infections developed in 14% (16/114) with an IV catheter-related infection, 15% (17/113) with a removable but non-IV catheter-related infection, 10% (25/249) with a non-removable focus of infection, and 7% (9/134) of those with no established focus. A metastatic focus of infection occurred more commonly in younger patients (p = 0.003) and in IDU (23% in IDU versus 9% non-IDU, p = 0.002).

The cumulative probability of hospital discharge by 14 and 30 days after positive blood culture was 28% (95% CI 24–32%) and 55% (51–59%) respectively. The estimated proportion discharged by 30 days was 42% (95% CI 33–50%) for MRSA bacteremia and 60% (95% CI 55–64%) for MSSA bacteremia.

### Outcome: inpatient mortality

Twenty-four percent (139/587) died during admission. Forty-five (32%) deaths occurred within 3 days of positive culture, 55 (40%) after 4–14 days, 35 (25%) after 15–60 days and only 4 (3%) after >60 days. 58% (80/139) of deaths were considered by the attending physicians to be directly attributable to SAB, similar for MRSA (62%) and MSSA (56%) bacteremia. All but one of the 16 deaths in Vietnam/Nepal were attributable to SAB compared to around half in the UK (65/123).

**Figure 2:**
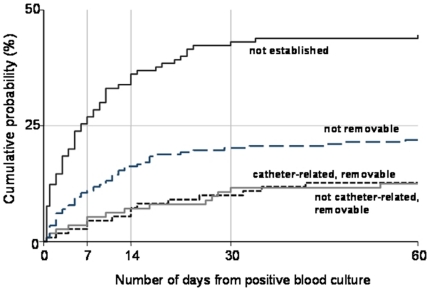
Cumulative incidence of in-patient mortality from date of positive blood culture, by focus of infection. X-axis truncated at 60 days since there were only 4 deaths occurring after this time point.

Risk of mortality varied significantly by focus of infection, being highest among patients with an undefined focus (**table 6**; **figure 2**). Death was also associated with older age and increasing duration from admission to positive blood culture. After adjustment for these factors we found no evidence of differences between patients with MRSA and MSSA bacteremia, nor between UK versus Vietnam/Nepal (**table 6**).

**Table 6:** Baseline factors associated with inpatient mortality following positive blood culture.

Factors	Number (%) died during admission	Univariable analyses^1^		Multivariable analyses^1^	
		Hazard Ratio (95% CI)	P-value	Hazard ratio (95% CI)	P-value
Overall	139/587 (24%)				
Country:					
UK	123/533 (23%)	1	0.21	1	0.15
Vietnam/Nepal	16/54 (30%)	1.42 (0.83–2.43)		1.60 (0.84–3.04)	
Susceptibility to methicillin:					
MSSA	100/456 (22%)	1	0.06	1	0.77
MRSA	37/125 (30%)	1.43 (0.98–2.07)		1.14 (0.77–1.69)	
Gender:					
Male	89/389 (23%)	1	0.56	1	0.94
Female	50/198 (25%)	1.11 (0.79–1.56)		1.01 (0.71–1.44)	
Age at positive blood culture result:					
<60 years	40/279 (14%)	1		1	
60–79 years	59/200 (30%)	2.21 (1.48–3.30)		2.15 (1.43–3.23)	
≥80 years	40/101 (40%)	3.17 (2.05–4.92)	<0.001	2.98 (1.88–4.73)	<0.001
Intravenous drug use:					
No	122/494 (25%)	1		1	
Yes	14/74 (19%)	0.74 (0.43–1.29)	0.29	1.41 (0.72–2.75)	0.31
Diabetes mellitus:					
No	107/448 (24%)	1		1	
Yes	23/116 (20%)	0.81 (0.51–1.26)	0.35	0.67 (0.42–1.06)	0.09
Immune-suppression					
No	70/290 (24%)	1		1	
Yes	60/253 (24%)	0.99 (0.71–1.40)	0.97	0.97 (0.68–1.38)	0.86
Days from admission to positive blood culture:					
Before/same day	55/306 (18%)	1		1	
1–6 days	35/151 (23%)	1.31 (0.86–2.00)		1.42 (0.92–2.19)	
7–13 days	21/54 (39%)	2.44 (1.48–4.02)		2.84 (1.72–4.71)	
≥14 days	28/76 (37%)	2.19 (1.41–3.42)	0.001	2.23 (1.37–3.62)	<0.001
Duration of symptoms before blood culture taken:					
≤24 hours	52/208 (25%)	1		1	
25–72	30/133 (23%)	0.83 (0.54–1.27)		0.97 (0.62–1.52)	
>72	26/140 (19%)	0.71 (0.45–1.13)	0.15	0.87 (0.50–1.53)	0.88
Focus of infection:					
Intra-venous catheter related only, and removable	16/110 (15%)	1		1	
Other source of infection, and removable	14/112 (13%)	0.86 (0.42–1.74)		1.06 (0.52–2.16)	
Not removable	51/228 (22%)	1.64 (0.95–2.84)		2.05 (1.18–3.57)	
Not established	58/130 (45%)	3.89 (2.26–6.69)	<0.001	4.17 (2.41–7.23)	<0.001

1 Hazard ratios of inpatient mortality were estimated using competing risks method, with hospital discharge a competing risk. Missing data for covariates were imputed using multiple imputation chained equation methods.

## Discussion

Opinions regarding the optimal treatment of SAB are divided [12], but the effects of this uncertainty on clinical practice and patient outcome are unknown. SAB is becoming increasingly important in lower income countries, but there is even less information available from these regions[20].

We found patients with SAB in Asia (predominantly Vietnam) were younger and more likely to be IDU and have endocarditis, than those in the UK, who were more likely to have IV catheter-related infections. These differences suggest a higher proportion of SAB in Vietnam/Nepal were community-acquired (not recorded in our study). The proportion of methicillin-resistant SAB was similar in the UK (21%) and Vietnam/Nepal (19%). A recent study from Thailand reported a higher proportion of methicillin-resistant SAB (28%), of which 78% were considered hospital-acquired[4], suggesting there may be substantial regional differences in the epidemiology of SAB in Asia. Further studies are required to determine the epidemiology of invasive *S. aureus* infections in this region.

Echocardiography is considered by some to be an essential component of the clinical assessment of all SAB[21], [22], yet was performed in only 50% of cases in the UK and 28% in Vietnam/Nepal. Echocardiography was performed in a surprisingly low proportion (45%) of patients in whom no focus was established. This may be explained by lack of availability in the Asian centres, or by the death of patients before echocardiography and other investigations could be performed (**figure 2**).

Failure to remove a removable infection focus is an independent risk factor for SAB treatment failure[23], [24]. 38% of infections were secondary to a removable focus (of which approximately half were IV catheter-related) and the proportion and speed of focus removal did not differ significantly between centres. In contrast, although the majority (81%) of patients started treatment within one day of the positive culture being taken, this proportion varied significantly between UK centres (**table 3**), as did the antibiotic regimens used. Contrary to current guidelines [8], [9], [10], [11], a third of UK patients received <14 days of intra-venous antibiotics, and 16% received <14 days treatment by any route. This is consistent with a recent survey of 266 European infection specialists: 35% reported they would use antibiotics for 7 days or less for the treatment of uncomplicated MRSA bacteremia and 10% said they would consider oral antibiotics for the initial treatment of MRSA bacteremia [12]. We found 25% of UK cases of SAB received more than half their treatment exclusively by the oral route, which varied from 12% and 40% between centres and that the proportion was higher if the isolate was methicillin-susceptible.

The choice of antibiotics used to treat SAB varied according to the susceptibility of the infecting organism, local preferences, and availability. Vancomycin was the favoured first-line glycopeptide for most cases of MRSA bacteremia, but two centres used teicoplanin almost exclusively, and one used linezolid in half of cases. Likewise, combination therapy was used to treat nearly all patients (94%) in some centres but few (14%) in others, and the choice of combinations varied widely. Similar proportions of patients with MRSA and MSSA bacteremia received combination therapy, but combinations were more likely to be used in patients with a non-removable focus. Rifampin was rarely used in Vietnam/Nepal, possibly because of the high prevalence of tuberculosis in these countries and the dangers of developing resistance in those with unsuspected active tuberculosis.

24% of patients died in hospital, which is similar to previous reports[3], [25], although just over half the deaths were directly attributed to the infection. Case fatality was similar in the UK (23%) and Vietnam/Nepal (30%), although the numbers of deaths in the later could have been under-estimated by the tendency in Vietnam for relatives to take seriously ill patients home to die. Indeed, one Vietnamese centre was excluded from the analysis as no in-patient deaths were recorded. Older age, an unidentified focus of infection, and longer duration between admission and bacteremia were independent risk factors for all-cause mortality (**table 6**). Others have reported age and focus to be strongly associated with outcome[24], [25], [26], but duration of admission prior to bacteremia has not been previously linked to outcome. This finding is likely explained by an association between prolonged admission and increased co-morbid conditions, but cannot be confirmed with the limited baseline clinical characteristics recorded. There was a trend for methicillin-resistance to be associated with in-hospital death by univariate analysis, but this association was not sustained in the multivariable analysis; others have reported similar findings[3], [25], [27] suggesting factors other than antibiotic resistance are the strongest determinants of outcome in MRSA bacteremia. In particular, we did not attempt to associate variations in treatment practices (for example, duration or route of antibiotic therapy) with outcome because of the strong likelihood of selection bias influencing the results. Such an analysis might produce misleading results with a potentially dangerous influence on clinical practice (wrongly justifying short or long durations of treatment, for example) and might obscure the pressing need to address the major questions surrounding the management of SAB through randomised controlled trials.

In summary, we have demonstrated that SAB is a serious infection in the UK and Vietnam, associated with high in-hospital mortality and significant variation in practice between the two regions and between centres in the UK. Adherence to published treatment guidelines was poor. In particular, a substantial proportion of patients with SAB received less than the recommended duration of therapy and antibiotic combinations were used far more widely than recommended. Until such time as the key questions in management have been addressed through large randomised controlled trials, extensive variations in practice will likely persist with uncertain consequences for patient care.

## Materials and Methods

Clinical data were recorded prospectively on all adults (>15 years) with *S. aureus* isolated from one or more blood culture. Patients were excluded if the blood isolate was mixed with another pathogen. Organisms were identified and antibiotic sensitivity testing performed according to standard methods by each centre's microbiology laboratory.

Data were collected between November 5^th^ 2008 and November 5^th^ 2009 in 8 centres in the UK and 4 in Asia (3 Vietnam, 1 Nepal) (see acknowledgements). One centre (Cambridge) joined the network in October 2009. All UK centres are large National Health Service hospitals, which provide secondary and tertiary referral care. Two centres in Vietnam (Hospital for Tropical diseases, Ho Chi Minh City; National Hospital for Tropical Diseases, Hanoi) provide secondary and tertiary referral services for infectious diseases; the third (Bach Mai Hospital, Hanoi) provides general medical facilities for the local population. Patan Hospital, Kathmandu, Nepal, provides primary and secondary healthcare facilities.

Physicians caring for the patients identified eligible patients and entered individual, anonymized data via a commercial web-based electronic data collection system (MACRO, *InferMed* limited, UK) with a full audit trail. All data were held on a central secure server. Data inconsistencies were monitored throughout the investigation by the statistician and principal investigator and returned to the centres for review and amendment if necessary.

The UK National Research Ethics Service reviewed the protocol and deemed the investigation an evaluation of service, not requiring review by an ethics committee. Each UK centre obtained permission from their Medical Director to collect the data. Separate permissions were obtained to undertake the investigation in Vietnam and Nepal from the Oxford Tropical Research Ethics Committee and the local institutional review boards.

### Statistical analysis

We compared factors relating to the management of SAB between regions, UK centres, focus of infection, and methicillin susceptibility. Differences between groups were assessed using chi-squared and Fisher's exact tests for binary variables, and Wilcoxon rank-sum and Kruskal-Wallis tests for continuous variables. Focus of infection was pre-specified as related to a removable central/peripheral intravenous catheter (without other source); related to another removable focus (e.g. drainable soft tissue abscess, removable medical device/prosthesis); related to an irremovable focus (e.g. skin/soft tissue infection without abscess, native heart valve, osteomyelitis); or the focus was not established. Patients who died on therapy or within 2 days of stopping therapy were excluded from analyses of duration of antibiotic therapy.

For analysis of inpatient mortality, only patients with date of positive blood culture and date of discharge or death available were included. One Vietnamese centre (27 patients) recorded no inpatient deaths and was excluded from this analysis: it is common practice for dying Vietnamese patients to receive terminal care from their families at home and this information was not recorded. When this occurred in the other Vietnamese centres, date of discharge was recorded as date of death. Follow-up of patients was considered from the date of positive blood culture until date of death or date of discharge. Since inpatient mortality and hospital discharge are competing events, applying standard survival analysis methods could lead to biased results[17]. Therefore, cumulative incidence of inpatient mortality and hospital discharge and hazard ratios were estimated using competing risks methods[17], [18]. The effects of the demographic and clinical factors at baseline on inpatient mortality were assessed. To avoid excluding patients with missing factors in regression models, missing data were imputed using chained equation methods with 20 imputations[19]. All analyses were undertaken using STATA version 11 (STATA Corporation, Texas, USA).
